# Analysis of oxygen uptake efficiency parameters in young people with cystic fibrosis

**DOI:** 10.1007/s00421-018-3926-8

**Published:** 2018-07-12

**Authors:** Owen W. Tomlinson, Alan R. Barker, Lucy V. Chubbock, Daniel Stevens, Zoe L. Saynor, Patrick J. Oades, Craig A. Williams

**Affiliations:** 1 Children’s Health and Exercise Research Centre, Sport and Health Science, College of Life and Environmental Sciences, University of Exeter, St Luke’s Campus, Heavitree Road, Exeter, EX1 2LU UK; 2 Royal Devon and Exeter NHS Foundation Trust Hospital, Barrack Road, Exeter, EX2 5DW UK; 3 Division of Respirology, Department of Pediatrics, Faculty of Medicine and School of Health and Human Performance, Faculty of Health Professions, Dalhousie University, Stairs House, 6230 South St., PO BOX 15000, Halifax, NS B3H 4R2 Canada; 4 Department of Sport and Exercise Science, Faculty of Science, University of Portsmouth, Spinnaker Building, Cambridge Road, Portsmouth, PO1 2ER UK; 5 Paediatric and Adult Respiratory Departments, University Hospital Southampton, Tremona Road, Southampton, SO16 6YD UK

**Keywords:** Adolescence, Aerobic fitness, Exercise testing, Paediatrics, Respiratory disease

## Abstract

**Purpose:**

This study characterised oxygen uptake efficiency (OUE) in children with mild-to-moderate cystic fibrosis (CF). Specifically, it investigated (1) the utility of OUE parameters as potential submaximal surrogates of peak oxygen uptake ($\dot {V}{{\text{O}}_{2{\text{peak}}}}$), and (2) the relationship between OUE and disease severity.

**Methods:**

Cardiopulmonary exercise test (CPET) data were collated from 72 children [36 CF, 36 age- and sex-matched controls (CON)], with OUE assessed as its highest 90-s average (plateau; OUEP), the gas exchange threshold (OUE_GET_) and respiratory compensation point (OUE_RCP_). Pearson’s correlation coefficients, independent *t* tests and factorial ANOVAs assessed differences between groups and the use of OUE measures as surrogates for $\dot {V}{{\text{O}}_{2{\text{peak}}}}$.

**Results:**

A significant (*p* < 0.05) reduction in allometrically scaled $\dot {V}{{\text{O}}_{2{\text{peak}}}}$ and all OUE parameters was found in CF. Significant (*p* < 0.05) correlations between measurements of OUE and allometrically scaled $\dot {V}{{\text{O}}_{2{\text{peak}}}}$, were observed in CF (*r* = 0.49–0.52) and CON (*r* = 0.46–0.52). Furthermore, measures of OUE were significantly (*p* < 0.05) correlated with pulmonary function (FEV_1%predicted_) in CF (*r* = 0.38–0.46), but not CON (*r* = −0.20–0.14). OUEP was able to differentiate between different aerobic fitness tertiles in CON but not CF.

**Conclusions:**

OUE parameters were reduced in CF, but were not a suitable surrogate for $\dot {V}{{\text{O}}_{2{\text{peak}}}}$. Clinical teams should, where possible, continue to utilise maximal CPET parameters to measure aerobic fitness in children and adolescents with CF. Future research should assess the prognostic utility of OUEP as it does appear sensitive to disease status and severity.

## Introduction

It is well established that a high level of aerobic fitness, typically characterised by peak oxygen uptake ($\dot {V}{{\text{O}}_{2{\text{peak}}}}$), is of benefit for young people with cystic fibrosis (CF). A higher $\dot {V}{{\text{O}}_{2{\text{peak}}}}$ is associated with an improved quality of life (Hebestreit et al. 2014), reduced risk of hospitalisation for pulmonary exacerbations (Pérez et al. 2014) and reduced mortality risk (Nixon et al. 1992; Pianosi et al. 2005). As a result, regular cardiopulmonary exercise testing (CPET) is recommended by the European CF Society and endorsed by the European Respiratory Society (Hebestreit et al. 2015), to monitor changes in aerobic fitness and guide decisions concerning clinical status and therapeutic interventions.

CPET is considered the gold standard method to assess aerobic fitness, with assessment of $\dot {V}{{\text{O}}_{2{\text{peak}}}}$ requiring the individual to provide a maximal physical effort. Factors such as excessive dyspnoea and/or a lack of motivation may cause individuals with CF to be unwilling or unable to reach volitional exhaustion and thus $\dot {V}{{\text{O}}_{2{\text{peak}}}}$. It has, therefore, been proposed that submaximal markers of aerobic fitness should be investigated as viable alternatives that can provide clinically useful information in such circumstances (Williams et al. 2014).

Previous research has shown the oxygen uptake efficiency slope (OUES) (Baba et al. 1996) to be a potentially useful submaximal parameter of aerobic fitness due to its high correlation with $\dot {V}{{\text{O}}_{2{\text{peak}}}}$ in clinical populations, including adults with CF (Gruet et al. 2010). However, there are several issues that preclude the use of OUES as an alternative marker of aerobic fitness in CF. First, OUES is dependent on body size and requires allometric scaling to normalise data (Tomlinson et al. 2017)—a process that may be time consuming in clinical practice. Second, the OUES has a high level of variability [as measured by coefficients of variation (CV)], both between participants, and in terms of test–retest reproducibility in healthy adults (Sun et al. 2012b) and children (Bongers et al. 2015). Finally, the OUES is unable to discriminate aerobic fitness within children and adolescents with mild-to-moderate CF (Williams et al. 2018).

The utility of other submaximal CPET parameters in children with CF, such as oxygen uptake efficiency (OUE)—the ratio between oxygen uptake ($\dot {V}{{\text{O}}_2}$) and ventilation (${\dot {V}_{\text{E}}}$) [$\dot {V}{{\text{O}}_2}$/${\dot {V}_{\text{E}}}$ (Sun et al. 2012b)]—therefore, warrants consideration. Unlike the OUES, which utilises a log-transformation of ${\dot {V}_{\text{E}}}$ (Baba et al. 1996) to linearise the non-linear ventilatory profile often observed during incremental exercise, the OUE parameter accommodates this curvilinear relationship between ${\dot {V}_{\text{E}}}$ and $\dot {V}{{\text{O}}_2}$ (Bongers et al. 2015). Furthermore, OUE has been shown to have less variability (CV) than OUES within groups of adults (39.5 vs. 14.6%) (Sun et al. 2012b) and children (32.9 vs. 10.9%) (Bongers et al. 2015) and is not dependent on body size (Sun et al. 2012b). This independence of body size, therefore, removes potential bias due to growth and the subsequent need to scale data, which may be of further benefit in a clinical setting.

Practically, OUE can be measured at any point during an incremental exercise test. However, the highest 90-second (s) plateau (oxygen uptake efficiency plateau; OUEP), which typically occurs prior to, or at, the ventilatory threshold (VT) (Bongers et al. 2015) or gas exchange threshold (GET) (Sun et al. 2012b), has been shown to be a predictor of mortality in heart failure (Sun et al. 2012a). Despite demonstrated clinical utility in cardiac populations, its role in chronic respiratory disease remains unknown. Furthermore, given that the ratio of ${\dot {V}_{\text{E}}}$ to $\dot {V}{{\text{O}}_2}$ (ventilatory equivalent for oxygen) at peak exercise has been shown to be a more significant predictor of mortality in children and adolescents with CF than body mass relative $\dot {V}{{\text{O}}_{2{\text{peak}}}}$ (Hulzebos et al. 2014), it is clear that the relationship between ${\dot {V}_{\text{E}}}$ and $\dot {V}{{\text{O}}_2}$ is of clinical significance, and warrants further investigation, particularly when it is not feasible nor possible to assess $\dot {V}{{\text{O}}_{2{\text{peak}}}}$, e.g. due to pathophysiological or motivational reasons. Therefore, the OUE (and in particular the OUEP) has the potential to be considered submaximal measures of aerobic fitness that could be used to quantify pathophysiological and/or therapeutically induced changes. However, evidence for this utilisation of OUE is required, with recent research calling for further investigation into the prognostic properties of other OUE parameters in children and adolescents with chronic health conditions, such as CF (Bongers et al. 2015).

Therefore, the aim of this study was to explore the utility of OUE parameters, in children and adolescents with mild-to-moderate CF, as potential submaximal surrogates for $\dot {V}{{\text{O}}_{2{\text{peak}}}}$. This is conducted first by characterising the OUE responses during CPET in children and adolescents with mild-to-moderate CF, compared with age- and sex-matched controls; second, by assessing the utility of OUE as an objective, submaximal surrogate for $\dot {V}{{\text{O}}_{2{\text{peak}}}}$ in this population; third, identifying the relationship between OUE parameters and disease status and severity in individuals with CF.

## Methods

### Participants

Data from 72 children and adolescents (36 with mild-to-moderate CF and 36 age- and sex-matched CON; 21 males per group; mean age 13.3 ± 2.8 years) were included in this study. Participant characteristics are presented in Table 1.

**Table 1:** Anthropometric, pulmonary function and exercise-related differences between CF and CON groups

Variable	CF	CON	*p* value	ES
Age (years)	13.4 (2.7)	13.2 (2.9)	0.77	0.08
Stature (cm)	155.6 (13.5)	159.1 (15.2)	0.32	0.24
Body mass (kg)	50.15 (15.46)	51.15 (14.49)	0.78	0.07
BMI (kg m^−2^)	20.28 (3.67)	19.91 (4.18)	0.70	0.09
BSA (m^2^)	1.46 (0.28)	1.49 (0.28)	0.65	0.11
FEV_1_ (L)*	2.46 (0.97)	2.96 (0.86)	0.07	0.55
FEV_1_ (%_predicted_)*	85.0 (20.0)	97.5 (10.6)	**0.004**	0.71
FVC (L)*	3.10 (1.14)	3.44 (1.02)	0.30	0.31
FVC (%_predicted_)*	92.7 (16.6)	98.6 (11.0)	0.18	0.39
MVV (L min^−1^)*	86.2 (34.0)	103.6 (30.0)	0.07	0.53
$\dot {V}{{\text{O}}_{2{\text{peak}}}}$ (L min^−1^)	1.74 (0.57)	2.03 (0.88)	0.09	0.39
$\dot {V}{{\text{O}}_{2{\text{peak}}}}$ (mL kg^−1^ min^−1^)	37.74 (7.74)	39.93 (10.70)	0.32	0.23
$\dot {V}{{\text{O}}_{2{\text{peak}}}}$ (mL kg^−0.86^ min^−1^)	74.62 (15.21)	84.94 (23.51)	**0.031**	0.52
Relative $\dot {V}{{\text{O}}_{2{\text{peak}}}}$ (%_predicted_)	83.3 (16.8)	87.8 (20.8)	0.32	0.24
GET (L min^−1^)	0.91 (0.28)	1.12 (0.54)	**0.035**	0.49
GET (% $\dot {V}{{\text{O}}_{2{\text{peak}}}}$)	53.4 (9.3)	55.0 (8.0)	0.42	0.18
HR_max_ (beats min^−1^)	182 (8)	185 (14)	0.30	0.26
${\dot {V}_{\text{Emax}}}$ (L min^−1^)	74.66 (35.62)	69.18 (33.45)	0.50	0.16
${\dot {V}_{\text{Emax}}}$ (% MVV)*	88.3 (30.4)	60.9 (23.3)	**0.001**	0.97
OUEP (mL L^−1^)	35.58 (5.40)	45.09 (5.78)	< **0.001**	1.70
OUE_GET_ (mL L^−1^)	34.08 (5.40)	43.24 (5.08)	< **0.001**	1.75
OUE_RCP_ (mL L^−1^)	29.49 (4.95)	35.15 (4.52)	< **0.001**	1.19
OUEP (%_predicted_)	83.2 (13.9)	105.7 (13.0)	< **0.001**	1.68

Measures are presented as mean (± SD). Significant mean differences are denoted by a bold *p* value. *Unequal groups for pulmonary variables: CF = 36, CON = 18

*BMI* body mass index, *BSA* body surface area, *FEV*_*1*_ forced expiratory volume in 1 s, *FVC* forced vital capacity, *MVV* maximal voluntary ventilation, $\dot {V}{O_2}$ volume of oxygen uptake, *GET* gas exchange threshold, *HR* heart rate, ${\dot {V}_E}$, minute ventilation, *RER* respiratory exchange ratio, *OUEP* oxygen uptake efficiency plateau, *OUE*_*GET*_ oxygen uptake efficiency at the gas exchange threshold, *OUE*_*RCP*_ oxygen uptake efficiency at the respiratory compensation point, *ES* effect size

### Ethics approval

This study was a retrospective analysis of existing data, and, therefore, did not require additional ethics approval. Ethics approval for original data collected was approved by the South West NHS Research Ethics Committee and the University of Exeter Sport and Health Sciences Ethics Committee. Fully informed written consent and assent were obtained from parents/guardians and paediatric participants, respectively.

### Anthropometric variables

Stature was measured to the nearest 0.001 m using a stadiometer (Holtain Ltd., Crymych, UK) and body mass to the nearest 0.01 kg using digital scales (Seca, Birmingham, UK). Body mass index (BMI) was subsequently calculated, and body surface area (BSA) was estimated using the Haycock equation (Haycock et al. 1978).

### Pulmonary function

Pulmonary function was assessed using flow-volume loop spirometry, with the maximal values from three acceptable manoeuvres for forced expiratory volume in 1 s (FEV_1_) and forced vital capacity (FVC) expressed relative to normative reference values from the Global Lung Function Initiative (Quanjer et al. 2012). Maximal voluntary ventilation (MVV) was calculated by multiplying FEV_1_ (L) by 35 (Wasserman et al. 2005).

### Exercise variables

All participants undertook a CPET to volitional exhaustion on an electronically braked cycle ergometer (Lode, the Netherlands) to determine maximal and submaximal measures of aerobic fitness. Breath-by-breath changes in pulmonary gas exchange and ventilation were measured, and subsequently averaged to 10-s time intervals. Of the 72 participants within the study, 33 children (20 CF, 13 CON) undertook a previously described supramaximal verification bout to determine a ‘true’ $\dot {V}{{\text{O}}_{2{\text{peak}}}}$ (Barker et al. 2011; Saynor et al. 2013a). However, as not all participants underwent this verification testing, the highest $\dot {V}{{\text{O}}_2}$ obtained during the course of testing procedures is referred to as ‘$\dot {V}{{\text{O}}_{2{\text{peak}}}}$’. Following determination of $\dot {V}{{\text{O}}_{2{\text{peak}}}}$, the GET and respiratory compensation point (RCP) were independently verified by two researchers using methods described by Beaver et al. (1986)—the disproportionate increases in $\dot {V}{\text{C}}{{\text{O}}_2}$ relative to $\dot {V}{{\text{O}}_2}$ (i.e. *V* slope method for GET) and ${\dot {V}_{\text{E}}}$ relative to $\dot {V}{\text{C}}{{\text{O}}_2}$ for RCP. This process is reliable in children with CF [CV = 11.2%, Saynor et al. (2013b)], and those without CF [CV = 7.5%, Fawkner et al. (2002)]. $\dot {V}{{\text{O}}_{2{\text{peak}}}}$ was compared to normative reference values, chosen due their similar participant characteristics and methodology, whilst also accounting for age and sex (Bongers et al. 2014a), and split into aerobic fitness tertiles [a division shown to predict mortality in CF (Pianosi et al. 2005)] for each group. Reliability of all gaseous exchange variables for children and adolescents with CF (Saynor et al. 2013b), and without CF (Welsman et al. 2005; Bongers et al. 2015), have previously been reported.

OUE values were calculated in line with previous work (Sun et al. 2012b), and were obtained by averaging $\dot {V}{{\text{O}}_2}$/${\dot {V}_{\text{E}}}$ in the 60 s prior to the GET (OUE_GET_) and RCP (OUE_RCP_). The OUEP was taken as the highest 90 s $\dot {V}{{\text{O}}_2}$/${\dot {V}_{\text{E}}}$ average. Warm-up and cool-down data during exercise were omitted from data analysis to isolate the incremental profile of the CPET. OUEP was also compared to normative values (Bongers et al. 2014a, 2015).

### Statistical analyses

Statistical analyses were performed using IBM SPSS Statistics v23 (IBM Corp., Armonk NY, USA). Allometric scaling was utilised to remove the influence of body mass from $\dot {V}{{\text{O}}_{2{\text{peak}}}}$ in both CF and CON groups (Welsman et al. 1996). Scaling of OUE variables was not required as there were no significant relationships with body size, thereby indicating size independence, as previously reported in adults (Sun et al. 2012b).

Pearson’s correlation coefficients determined relationships between all OUE parameters and $\dot {V}{{\text{O}}_{2{\text{peak}}}}$_,_ as well as, the traditional clinical marker of disease severity, FEV_1_ (expressed as a percentage of predicted). Independent sample *t* tests established mean differences in anthropometric, pulmonary function and CPET parameters between groups. Factorial analyses of variance (ANOVAs) were used to establish interaction effects between disease status and aerobic fitness tertiles (as described in ‘Exercise variables’) upon $\dot {V}{{\text{O}}_{2{\text{peak}}}}$ and OUE parameters. For ANOVAs, the tertiles for $\dot {V}{{\text{O}}_{2{\text{peak}}}}$ to which participants were categorised (i.e. high, middle, low) remained the same throughout all ANOVAs, regardless of OUE value. Where significant effects occurred, planned pairwise comparisons with a Sidak correction factor were applied, chosen for its correction of multiple comparisons (reducing Type 1 error), whilst simultaneously being less conservative than Bonferroni corrections (thus reducing Type 2 error) (Abdi 2007). Statistical significance was set at an alpha level of 0.05, and effect sizes (ES) for mean comparisons were described using Cohen’s thresholds (small = 0.2, medium = 0.5, large = 0.8) (Cohen 1992).

## Results

### Differences in OUE between groups

**Fig. 1:**
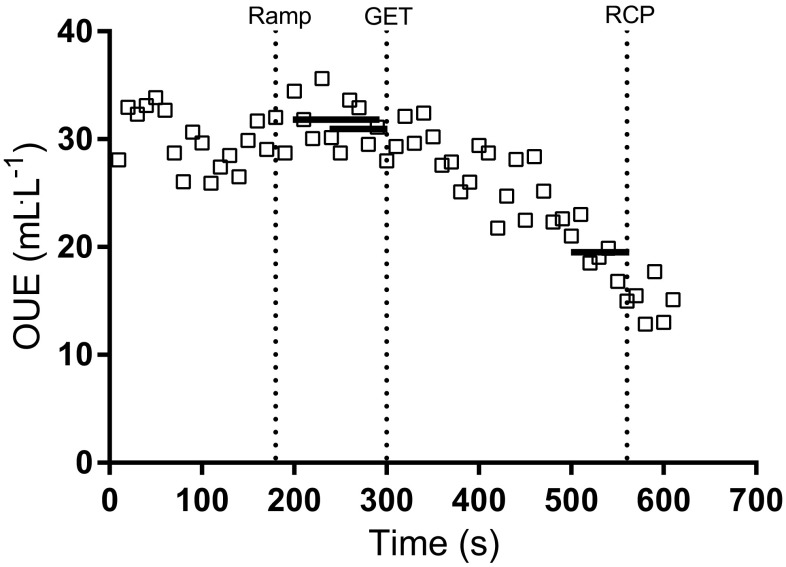
Profiles of OUEP, OUE_GET_ and OUE_RCP_ in a representative CPET from an individual child with CF (female, 12 years, homozygous ΔF508, FEV_1_ 82.0%_predicted_, $\dot {V}{{\text{O}}_{2{\text{peak}}}}$ 36.5 mL kg^−1^.min^−1^, 73.26 mL kg^−0.86^.min^−1^). Vertical line at 180 s indicates end of warm-up, and beginning of ramp phase. Vertical lines also indicate point of GET and RCP. Horizontal lines between 200–290 s = OUEP (31.9 mL L^−1^), 240–300 s = OUE_GET_ (31.0 mL L^−1^), 500–560 s = OUE_RCP_ (19.7 mL L^−1^)

**Fig. 2:**
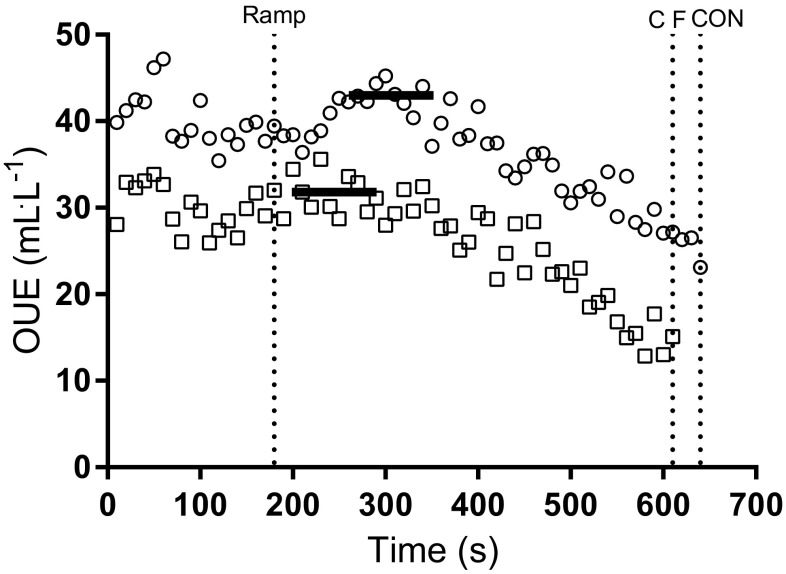
Differences in OUE ($\dot {V}{{\text{O}}_2}$/${\dot {V}_{\text{E}}}$) between two representative children, CF (open square) and CON (open circle), throughout a ramp incremental CPET. Vertical line at 180 s indicates the end of the warm-up and beginning of ramp phase of the test. Vertical lines at 610 and 640 s indicate exhaustion for CF and CON participants, respectively. Solid horizontal lines at 31.9 mL L^−1^ (CF) and 43.0 mL L^−1^ (CON) indicate OUEP (highest 90 s average) for each group, respectively

All OUE outcomes were detected in 68/72 participants (94%). Both OUE_GET_ and OUE_RCP_ were identified in 35/36 (97%) of children and adolescents in the CF group. In the CON group, OUE_GET_ was detected in all participants (36/36, 100%), and OUE_RCP_ was detected in 34/36 (94%) of participants. The profiles of OUEP, OUE_GET_ and OUE_RCP_ during a CPET for a representative individual with CF are shown in Fig. 1. A representative comparison of OUEP for one participant with CF against CON is shown in Fig. 2.

**Fig. 3:**
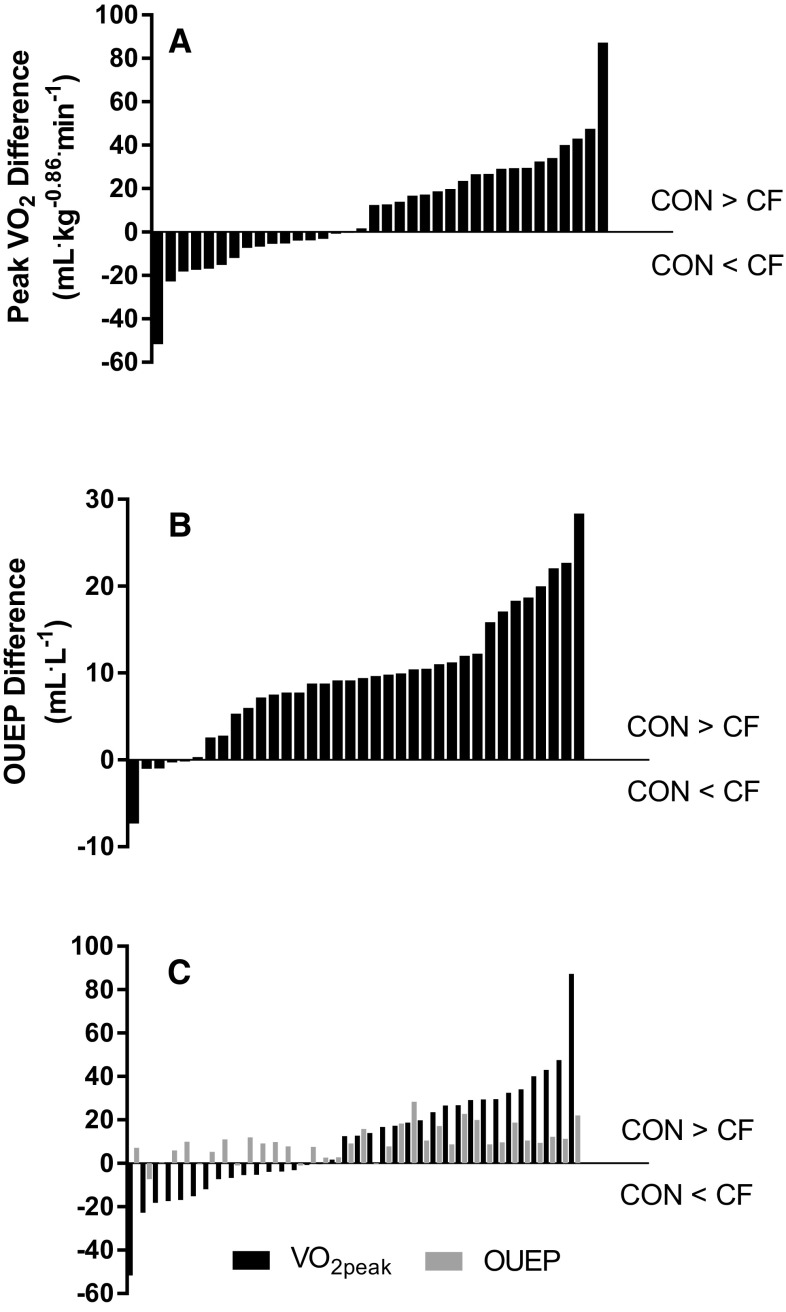
Individual differences between age- and sex-matched CON and CF pairs for CPET-derived variables. All plots are calculated as CON minus CF, i.e. bars underneath *y* = 0 on *x*-axis indicate participant with CF has a greater value than CON counterpart. **a** Differences in allometrically scaled $\dot {V}{{\text{O}}_{2{\text{peak}}}}$ between pairs. **b** Differences in OUEP between pairs, independent of differences in $\dot {V}{{\text{O}}_{2{\text{peak}}}}$. **c** Differences in $\dot {V}{{\text{O}}_{2{\text{peak}}}}$ (mL kg^−0.86^.min^−1^) between pairs, plotted alongside within-pair differences in OUEP (mL L^−1^). Black bars represent $\dot {V}{{\text{O}}_{2{\text{peak}}}}$ and grey bars indicate OUEP

Differences between groups were observed for pulmonary function, absolute GET, OUE_GET_, OUE_RCP_ and OUEP, with CON being significantly higher than CF. No significant difference was observed between CF and CON groups for $\dot {V}{{\text{O}}_{2{\text{peak}}}}$, when expressed as an absolute value. However, the CON group was revealed to have a significantly (*p* < 0.05) greater $\dot {V}{{\text{O}}_{2{\text{peak}}}}$ when allometric scaling had removed residual effects of body size (Table 1). Individual differences between age- and sex-matched pairs for $\dot {V}{{\text{O}}_{2{\text{peak}}}}$ and OUEP are displayed in Fig. 3.

### Correlation of with OUE with $\dot {V}{{\text{O}}_{2{\text{peak}}}}$

OUEP and OUE_GET_ were significantly and positively correlated with absolute $\dot {V}{{\text{O}}_{2{\text{peak}}}}$ in the CF and CON groups; however, OUE_RCP_ was not correlated with absolute $\dot {V}{{\text{O}}_{2{\text{peak}}}}$ in either CF or CON groups. OUEP and OUE_GET_, but not OUE_RCP_, were correlated with allometrically scaled $\dot {V}{{\text{O}}_{2{\text{peak}}}}$ in both CF and CON (Table 2).

**Table 2:** Correlations between OUE parameters and $\dot {V}{{\text{O}}_{2{\text{peak}}}}$, and FEV_1_

	CF	CON	Combined
Absolute $\dot {V}{{\text{O}}_{2{\text{peak}}}}$ (L min^−1^)			
OUE_GET_	**0.36 (0.036)**	**0.40 (0.017)**	**0.41 (**< **0.001)**
OUE_RCP_	0.12 (0.50)	0.29 (0.09)	**0.28 (0.022)**
OUEP	**0.43 (0.010)**	**0.42 (0.010)**	**0.44 (**< **0.001)**
OUEP (% predicted)	0.22 (0.20)	0.12 (0.51)	**0.24 (0.040)**
Allometrically scaled $\dot {V}{{\text{O}}_{2{\text{peak}}}}$ (mL kg^−0.86^.min^−1^)			
OUE_GET_	**0.49 (0.003)**	**0.46 (0.005)**	**0.51 (**< **0.001)**
OUE_RCP_	0.31 (0.08)	0.24 (0.17)	**0.35 (0.003)**
OUEP	**0.52 (0.001)**	**0.52 (0.002)**	**0.54 (**< **0.001)**
OUEP (% predicted)	**0.49 (0.003)**	**0.38 (0.021)**	**0.47 (**< **0.001)**
FEV_1_ (%_predicted_)*			
OUE_GET_	**0.38 (0.026)**	− 0.06 (0.83)	**0.44 (0.001)**
OUE_RCP_	0.07 (0.68)	0.14 (0.61)	0.24 (0.08)
OUEP	**0.43 (0.010)**	− 0.20 (0.43)	**0.43 (0.001)**
OUEP (% predicted)	**0.46 (0.005)**	− 0.19 (0.45)	**0.44 (0.001)**

Values are presented as correlation coefficients (*r*) with *p* values in parentheses. Bold text indicates a significant (*p* < 0.05) coefficient

$\dot {V}{O_2}$ oxygen uptake, *FEV*_*1*_ forced expiratory volume in 1 second

*Unequal samples for pulmonary variables: CF = 36, CON = 18

### Differences between aerobic fitness groups

When the data were split by tertiles according to allometrically scaled $\dot {V}{{\text{O}}_{2{\text{peak}}}}$, a significant difference in aerobic fitness was observed between tertiles within both CF (high: 91.0 ± 8.4 vs. mid: 74.7 ± 4.6 vs. low: 58.1 ± 7.5 mL kg^−0.86^.min^−1^) and CON (110.1 ± 16.3 vs. 86.0 ± 7.3 vs. 62.0 ± 12.5 mL kg^−0.86^.min^−1^) groups (*p* < 0.05 for all pairwise comparisons, ES = 1.91–4.13). However, when comparisons were made between groups, a significant difference in allometrically scaled $\dot {V}{{\text{O}}_{2{\text{peak}}}}$ between CF and CON was only evident in the high (*p* < 0.001, ES = 1.47) and middle (*p* = 0.50, ES = 1.85) aerobic fitness tertiles, not for the lowest (*p* = 0.39, ES = 0.38).

**Fig. 4:**
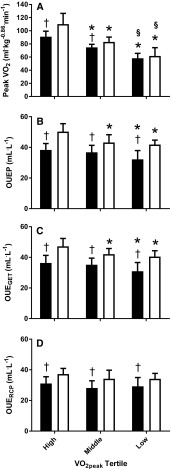
Comparison of $\dot {V}{{\text{O}}_{2{\text{peak}}}}$ (**a**) and OUE parameters (**b** OUEP; **c** OUE_GET_; **d** OUE_RCP_) between CF (black) and CON (white), split by $\dot {V}{{\text{O}}_{2{\text{peak}}}}$ tertile. *Significant (*p* < 0.05) difference from highest tertile (within group). ^§^Significant (*p* < 0.05) difference from middle tertile (within group). ^†^Significant (*p* < 0.05) difference between groups (within tertile)

When assessing OUEP by fitness tertile and disease group, significant main effects were seen for group (*p* < 0.001) and fitness tertile (*p* < 0.001), but no significant fitness tertile by group interactions were evident (*p* = 0.20; Fig. 4). Pairwise comparisons identified mean differences between CF and CON for OUEP at each level of fitness, respectively (high; 38.32 ± 4.21 vs. 50.26 ± 5.22, *p* < 0.001, ES = 2.52, middle; 36.22 ± 4.57 vs. 43.19 ± 5.06, *p* = 0.001, ES = 1.45, low; 32.19 ± 5.73 vs. 41.81 ± 2.93, *p* < 0.001, ES = 2.11).

Mean differences for OUEP were found between the highest and lowest between aerobic fitness tertiles within CF (*p* = 0.006, ES = 1.22), but not between high and middle (*p* = 0.62, ES = 0.48) nor middle and low (*p* = 0.11, ES = 0.78). Further pairwise comparisons revealed differences within the CON group, as the tertile with the highest aerobic fitness had a significantly higher OUEP than the middle (*p* = 0.001, ES = 1.38) and lowest (*p* < 0.001, ES = 2.00) tertiles. No significant difference was evident between the middle and lowest tertiles with regard to OUEP for CON (*p* = 0.85, ES = 0.33; Fig. 4).

When ANOVAs were repeated for OUE_GET_ and OUE_RCP_, significant main effects for group were found (*p* < 0.001) for both parameters. Further significance (*p* < 0.05) between groups was identified when split by fitness tertile. A main effect for fitness tertile was present for OUE_GET_ (*p* < 0.001), but not OUE_RCP_ (*p* = 0.08). Main interaction effects between group and aerobic fitness were not present for either OUE_GET_ (*p* = 0.34) or OUE_RCP_ (*p* = 0.88; Fig. 4). However, for OUE_GET_, pairwise comparisons revealed significant differences within CF between high and low fitness tertiles (*p* = 0.021, ES = 1.01). For CON, differences were found between high and low (*p* = 0.002, ES = 1.48), and high and middle fitness tertiles (*p* = 0.026, ES = 1.15). For OUE_RCP_, there were no differences between tertiles within groups (*p* = 0.37–1.00, ES = 0.01–0.86). In addition, pairwise comparisons revealed significant differences (*p* < 0.05) between groups at each tertile for both OUE_GET_ and OUE_RCP_ (Fig. 4).

### Relationship with disease severity (FEV_1_)

OUEP and OUE_GET_ were significantly correlated with FEV_1_ within the CF group, but OUE_RCP_ was not. None of the OUE parameters were significantly correlated with FEV_1_ within CON (Table 2). Allometrically scaled $\dot {V}{{\text{O}}_{2{\text{peak}}}}$ was significantly correlated with FEV_1_ (%_predicted_) in CF (*r* = 0.46, *p* = 0.004), but not CON (*r* = − 0.20, *p* = 0.43).

## Discussion

In this study, whilst all OUE parameters were significantly reduced in children and adolescents with CF in the current study, results show that OUE does not provide a viable surrogate for $\dot {V}{{\text{O}}_{2{\text{peak}}}}$ in this group. However, the novel finding of this study is that OUE appears to hold clinical utility as an independent marker of aerobic fitness, since it can differentiate between CF and CON, and holds a significant relationship with disease severity (as shown by FEV_1_) in the CF group. An example is shown in Fig. 3, whereby allometrically scaled $\dot {V}{{\text{O}}_{2{\text{peak}}}}$ was greater in individuals with CF in 16/36 (44%) age- and sex-matched pairs, but OUEP was only greater in individuals with CF in 5/36 (14%) matched pairs [and $\dot {V}{{\text{O}}_{2{\text{peak}}}}$ and OUEP were only greater in CF in 4/36 (11%) of cases], thus indicating reduced OUE in CF, regardless of fitness status. This is further corroborated by the significant relationship between OUE (OUEP, OUE_GET_) and FEV_1_ (%_predicted_) within the CF cohort, showing that OUE is associated with traditional clinical markers of disease severity.

For individuals with CF, a reduced aerobic fitness is a hallmark of disease progression (Orenstein and Higgins 2005) and assessment of $\dot {V}{{\text{O}}_{2{\text{peak}}}}$ is, therefore, recommended on at least an annual basis (Hebestreit et al. 2015). However, as maximal testing may not always be possible in this patient group (due to pathophysiological and/or motivation related factors), viable submaximal measures are needed to assess aerobic fitness. Whilst submaximal physiological thresholds such as the GET are related to disease severity (Thin et al. 2002), detection rates are variable in CF [12/13; 92% (Saynor et al. 2013b)], and non-CF [45/55; 82% (Hebestreit et al. 2000)] groups and are typically dependent on knowledge of $\dot {V}{{\text{O}}_{2{\text{peak}}}}$ to be expressed as a percentage of maximal capacity. In the present study, all OUE values were identified in the majority (94%) of participants, with OUEP identified in 100% of participants. The identification of OUEP is related to the averaging of 90 s of data and is not dependent on prior detection of the GET or RCP (to produce OUE_GET_ and OUE_RCP_). The OUEP occurs at a submaximal point near the VT (Bongers et al. 2015) and/or GET (Sun et al. 2012b), a threshold that reportedly occurs at 50–60% of $\dot {V}{{\text{O}}_{2{\text{peak}}}}$ in children and adolescents with CF (Bongers et al. 2014b; Saynor et al. 2014, 2016). Therefore, the exercise intensity required to generate a value for OUEP should be feasible for most children to achieve despite being unable or unwilling to exercise to exhaustion, such as those with advanced pulmonary disease, or more prone to increased levels of dyspnoea and desaturation upon exertion. The simplicity of the OUEP measure highlights how feasible a measure it may be to implement in busy clinical environments, suiting patients, researchers and clinicians alike.

In the current study, OUE variables were significantly correlated with $\dot {V}{{\text{O}}_{2{\text{peak}}}}$ in the CON and CF groups, indicating the two variables have a medium [as defined by Cohen (1992)] relationship (*R*^2^ = 27% between OUEP and allometrically scaled $\dot {V}{{\text{O}}_{2{\text{peak}}}}$ in both CF and CON). Given previous research (Williams et al. 2018) has identified differences in $\dot {V}{{\text{O}}_{2{\text{peak}}}}$ within, and between, CF and CON groups when split by aerobic fitness tertile, a division shown to predict for mortality (Pianosi et al. 2005), it would, therefore, be anticipated that parameters of OUE would follow a similar pattern in discriminating between individuals’ of differing aerobic fitness statuses. Differences are seen within the CON group for OUEP, with the highest fitness tertile having significantly greater OUEP relative to children in the middle and lowest fitness tertiles, thus, showing that OUEP can discriminate between individuals on different fitness status. However, the same discriminatory ability is not seen for the CF group as it is only the group with the lowest aerobic fitness that is different to the group with the highest fitness (Fig. 4). Therefore, despite a relationship with $\dot {V}{{\text{O}}_{2{\text{peak}}}}$, the inability to discriminate between the fitness groups shows that the OUEP cannot act as a surrogate for $\dot {V}{{\text{O}}_{2{\text{peak}}}}$.

Of the limited research to have characterised the OUEP in youth, a large cross-sectional study of 214 healthy Dutch children identified similar mean values for OUEP (boys, 42.6 ± 4.7; girls, 42.3 ± 4.6 mL L^−1^) and OUE at the VT (boys, 42.0 ± 4.6; girls, 41.9 ± 4.7 mL L^−1^) to those of the CON group in the current study (Bongers et al. 2015). They also identified a stronger relationship (*r* = 0.65, *p* < 0.01) between the OUEP and absolute $\dot {V}{{\text{O}}_{2{\text{peak}}}}$ than the CON group in the current study, potentially due to the higher ${\dot {V}_{{\text{Emax}}}}$ observed in both boys and girls (80 ± 25; 71 ± 21 L min^−1^ respectively) relative to the current CON group (69.2 ± 33.5 L min^−1^), which may, therefore, bias the relationship between $\dot {V}{{\text{O}}_2}$ and $\dot {V}{{\text{O}}_2}$/${\dot {V}_{\text{E}}}$ (OUE). However, as the current study builds upon this previous work and is the first to comprehensively examine OUE at multiple metabolic thresholds in children and adolescents with CF, only limited comparisons can be made, as no previous research has provided values against which to compare our novel data. Furthermore, the only application of OUE in clinical groups has been in adults with heart failure (Sun et al. 2012a), pulmonary hypertension (Tan et al. 2014), chronic obstructive pulmonary disease (Barron et al. 2016) and pulmonary embolism (Guo et al. 2016). However, minimal comparisons and inferences can be made against children with CF and these adult-onset, and predominantly vascular conditions.

As the current study has shown that OUEP (nor any OUE parameter) is not able to act as a surrogate measure of aerobic fitness, alternative submaximal factors must be considered. Ventilatory drive (${\dot {V}_{\text{E}}}$/$\dot {V}{\text{C}}{{\text{O}}_2}$) has received recent attention in predictive models of mortality (Hulzebos et al. 2014), and may be a viable candidate, given its low variability compared to ${\dot {V}_{\text{E}}}$/$\dot {V}{{\text{O}}_2}$ (Sun et al. 2002) and superior prognostic value relative to OUES in patients with heart failure (Arena et al. 2007). As such, further research should continue to explore the potential utility of this variable in individuals with CF, either as an alternative for $\dot {V}{{\text{O}}_{2{\text{peak}}}}$, or an independent prognostic variable. However, it is unclear whether any parameter of OUE may be of use in individuals with a more severe form of CF, or have longitudinal relevance in mild-to-moderate CF and, therefore, further research is warranted.

A number of limitations associated with the present study are worthy of comment. Primarily, this study is focused in children and adolescents with mild-to-moderate CF (FEV_1_ > 40%_predicted_). However, defining severity on FEV_1_ alone does not account for the nutritional measures, number of exacerbations, inflammatory markers and infection statuses that also contribute towards a patient profile and definition of severity. Consequently, these results may not be applicable to those with lower lung function, a cohort for whom FEV_1_ has a greater influence upon $\dot {V}{{\text{O}}_{2{\text{peak}}}}$ (Pastre et al. 2014). Furthermore, the CON group in the current study displays a reduced level of aerobic fitness relative to previous studies investigating OUE (Bongers et al. 2015), which may explain the number of individuals with CF having a higher $\dot {V}{{\text{O}}_{2{\text{peak}}}}$ within age- and gender-matched pairs (Fig. 3). In addition, the lack of all participants undertaking supramaximal verification bouts (Barker et al. 2011) within CPETs could potentially influence detection of a ‘true’ $\dot {V}{{\text{O}}_{2\hbox{max} }}$ (hence our use of $\dot {V}{{\text{O}}_{2{\text{peak}}}}$). This is likely to have minimal effect, as previous work has shown that the ramp-only test elicits a ‘true’ $\dot {V}{{\text{O}}_{2{\text{max}}}}$ in ~ 90% of healthy children (Barker et al. 2011) and ~ 80% of children with CF (Saynor et al. 2013a). Finally, when these methodological issues are considered in conjunction with our sample size, true effects may be obscured regarding the ability for OUEP to discriminate aerobic fitness. For example, the difference between middle- and low-fitness tertiles in CF revealed a *p* value of 0.11, yet an ES of 0.78, thus indicating an effect is likely present but cannot be statistically confirmed. We have utilised the Sidak correction factor in this study as opposed to the more conservative Bonferroni in an attempt to alleviate the potential for Type 2 errors, yet statistical significance was not found in some comparisons and a statistical error might still have occurred. Larger clinical sample sizes would be advantageous but are not always feasible in young people who are sick.

## Conclusion

The current study is the first to comprehensively characterise parameters of OUE in children and adolescents with mild-to-moderate CF, and assess the utility of such parameters as submaximal surrogates for $\dot {V}{{\text{O}}_{2{\text{peak}}}}$. Despite promising findings in other clinical populations, and a significant relationship between OUE and allometrically scaled $\dot {V}{{\text{O}}_{2{\text{peak}}}}$ in the present study, the inability to differentiate between aerobic fitness statuses indicates that OUE is unable to provide a viable surrogate for $\dot {V}{{\text{O}}_{2{\text{peak}}}}$ in this population. Further research is, therefore, warranted to identify suitable submaximal variables to characterise aerobic fitness status in children and adolescents with CF when determination of $\dot {V}{{\text{O}}_{2{\text{peak}}}}$ is not possible. Moreover, the prognostic utility of OUE in CF when $\dot {V}{{\text{O}}_{2{\text{peak}}}}$ cannot be determined also warrants investigation.

## Abbreviations

CF: Cystic fibrosis
CON: Control
CPET: Cardiopulmonary exercise test
ES: Effect size
FEV_1_: Forced expiratory volume in 1 s
FVC: Forced vital capacity
GET: Gas exchange threshold
OUE: Oxygen uptake efficiency
OUEP: Oxygen uptake efficiency plateau
OUE_GET_: Oxygen uptake efficiency at the gas exchange threshold
OUE_RCP_: Oxygen uptake efficiency at the respiratory compensation point
OUES: Oxygen uptake efficiency slope
RCP: Respiratory compensation point
$\dot {V}{{\text{O}}_2}$: Volume of oxygen uptake
